# National Survey Highlights the Urgent Need for Standardisation of Embryo Transfer Techniques in the UK

**DOI:** 10.3390/jcm10132839

**Published:** 2021-06-27

**Authors:** Lewis Nancarrow, Nicola Tempest, Andrew J. Drakeley, Roy Homburg, Richard Russell, Dharani K. Hapangama

**Affiliations:** 1 Centre for Women’s Health Research, Department of Women’s and Children’s Health, Institute of Life Course and Medical Sciences, University of Liverpool, Member of Liverpool Health Partners, Liverpool L8 7SS, UK; ntempest@liverpool.ac.uk (N.T.); dharani@liv.ac.uk (D.K.H.); 2 Hewitt Centre for Reproductive Medicine, Liverpool Women’s NHS Foundation Trust, Liverpool L8 7SS, UK; andrew.drakeley@lwh.nhs.uk (A.J.D.); Richard.Russell@lwh.nhs.uk (R.R.); 3 Liverpool Women’s NHS Foundation Trust, Liverpool Health Partners, Liverpool L8 7SS, UK; 4 Homerton Fertility Unit, Homerton University Hospital, Homerton Row, London E9 6SR, UK; roy.homburg@gmail.com

**Keywords:** embryo transfer, survey, standardisation, IVF, in vitro fertilisation

## Abstract

Embryo transfer (ET) is one of the vital steps in the in vitro fertilisation (IVF) process, yet there is wide variation in ET technique throughout the UK, without a nationally approved standardised approach. The aim of this study was to gain contemporaneous information regarding the current clinical ET practice in the UK. Method: A 38-question electronic survey was distributed to the 79 UK Human Fertilisation and Embryology Authority (HFEA) registered clinics performing ETs. Results: In total, 59% (47/79) of units responded, 83% (39/47) performing ultrasound-guided transfers, with 42% (20/47) of units using a tenaculum; 22% (10/45) would proceed with transfer regardless of fluid in the endometrial cavity. In 91% (43/47) of units, embryos were deposited in the upper/middle portion of the uterine cavity, but interpretation of this area ranged from 0.5 to >2 cm from the fundus, with 68% (32/47) allowing patients to mobilise immediately after transfer. In 60% (27/45) of clinics, success rates were based on clinical pregnancy rates (CPR). Conclusion: Within the UK there is a wide range of variability in ET techniques, with >70% of discordance in survey-responses between clinics. Whilst there are areas of good practice, some disadvantageous techniques continue to persist. This survey emphasises the importance of developing a standardised, evidence-based approach to improve ET success rates.

## 1. Introduction

Transferring a good quality embryo in to an appropriately prepared uterine cavity is an integral part of the in vitro fertilisation (IVF) process and a fundamental step in conception [1]. Reproductive medicine as a speciality, and the IVF process in particular, have seen significant changes over the past 40 years, with many developments in both clinical practice and laboratory procedures [2]. However, during this time, there has been little change in the embryo transfer (ET) technique originally developed by Steptoe et al. [3,4] other than ultrasound guidance and the use of catheters specific for ET [2].

The best ET technique would deliver the embryo to the optimum location within the uterine cavity in the least traumatic way without disturbing the primed uterine environment [4]. The first described ET technique introduced and delivered a preloaded embryo with a soft catheter into the uterine cavity via the cervical canal [3]. The intrauterine position of the catheter tip for embryo deposition was either determined by measuring 6 cm from the external cervical os or by measuring the cavity length with a dummy transfer prior to the actual ET [1]. The first ultrasound-guided ET was reported in 1985 [5], and 30 years later a Cochrane review concluded that ultrasound guidance should be the recommended and preferred method for ET [1]. Despite this Cochrane guidance, a lack of universal implementation exists, demonstrated by two recent surveys showing wide variation in ET techniques [4,6]. The reason for this is thought to be multifactorial, with most of the published data on efficacy of ET techniques being conflicting, inconclusive or affected by confounding variables dependent on either the practitioner or the technique [1,4,7,8,9,10]. This is an important issue in IVF research. For example, studies using different embryo deposition points of 1, 1.5 or 2 cm from the fundus, and measuring the outcome of clinical pregnancy are confounded by the embryo deposition site [1,10,11,12,13]. Another example of conflicting evidence is the removal of cervical mucus prior to ET. Some studies recommend removal [14,15,16,17], whilst others, including a meta-analysis, failed to show any significant benefit [18,19]. Use of a patient relaxant, direction of the removal of the ET catheter and duration of bedrest following transfer are some of the other discordances between studies [4]. Such differences could also impact the outcomes between trials [20], resulting in misinterpretation of the available evidence. It is estimated that up to 30% of all cycle failures can be considered due to poor practice used in the transfer technique [21], and it has been shown that pregnancy rates can differ depending on the clinician performing the transfer [17], which emphasises the expertise required for this often-overlooked component of the IVF process [2].

The lack of consensus that exists at the present time may also be due to the apparent absence of a robust, specific guideline highlighting the practice of the ET technique. Such guidelines from professional organisations such as the British Fertility Society (BFS) or the European Society of Human Reproduction and Embryology (ESHRE) may facilitate standardisation of best evidence-based practice, which is a fundamental first step towards improving clinical outcomes in IVF.

The last UK survey on ET was conducted nearly two decades ago, and their main recommendation was the need for a standardised national protocol to be implemented for ET [22]. Since then, new evidence has found that subtle differences between individual practitioners can significantly affect ET success rates despite using a similar technique [22,23,24]. Examples for these include two separate Cochrane reviews recommending the use of ultrasound guidance, as well as the use of soft catheters for ETs [1,25]. However, a universally available, standardised, national guideline or protocol for practitioners in IVF units in the UK is yet to be produced. Our aim, therefore, was to evaluate and gain insight into the current clinical practice regarding ET in the UK. Our data aims to provide the basis for future attempts to harmonise the practice in the UK with the formulation of a standardised protocol.

## 2. Materials and Methods

### 2.1. The Survey

Items in the survey were identified based on a literature review and expert clinical opinion. Expert clinical opinion was sought initially from local practitioners (reproductive medicine specialists and embryologists) at the Hewitt Fertility Centre, Liverpool, which is one of the larger National Health Service (NHS) IVF units in the UK with approximately 1800 fresh IVF/ICSI cycles being performed per annum. The initial survey questions were formulated in August 2018 after reviewing current ET techniques and by considering the practice pertinent to individual practitioners. The initial 33 question survey was subsequently modified after being peer reviewed by five other fertility specialists who were directly contacted by the authors, from IVF units around the country, before a final 38 question national survey was finalized and distributed to all IVF units in the country (Supplementary Figure S1). 

The survey questions were informed by current evidence relating to different aspects of the ET technique. The questions in the final survey included demographic information on the unit (type of practice, number of ETs per year, location) and important outcome measurements (including biochemical pregnancy rate (BPR), clinical pregnancy rates (CPR) and live birth rates (LBR)). We also included questions relevant to the ET technique (such as the type of catheter used, the use of ultrasound guidance, how practitioners clean the cervix) and questions relevant to the practitioners involved during the ET (which professionals were involved and their experience). Previous evidence suggested that the use of ultrasound guidance [1,7], soft catheters [25,26] and removal of cervical mucus [16] can improve ET success rates, and physician-associated factors also play an important role [27], thus these were included. Our data, therefore, provides evidence for heterogeneity in practice that may affect outcomes of clinical trials in this area, as well as highlighting existing uncertainties to focus on in future research efforts.

The final electronic survey was emailed on the 16 December 2018 through SurveyHero (www.surveyhero.com) to all clinical leads in the 79 Human Fertilisation and embryology authority (HFEA) registered units that perform ETs in the UK. SurveyHero is an online anonymous survey tool, and no patient-identifiable data were collected. Electronic reminders were sent out in the interim six-month period when they were requested to respond. When there was no response from clinical leads, other consultants within the same unit were contacted requesting a response to the survey. To remove duplication or inaccuracy of responses from a particular unit, the name of the organisation was included. If multiple responses were received from the same unit, the first response from that unit (after confirming concordance with duplicate responses) was used in the analysis.

As this was an anonymous survey with no patient-identifiable data, ethical approval was not required. 

### 2.2. Statistical Analysis

This survey was not designed as a comparative study or powered to detect differences. Therefore, in line with our research aims of the current national practice in the UK, we report summary statistics of the data obtained from the survey. Where possible, the Statistical package for the Social Sciences (SPSS) for Windows (Version 26; IBM Corporation, Chicago, IL, USA) was used to analyse categorical data using the χ^2^ test or the Student’s paired *t*-test for continuous data.

## 3. Results

**Figure 1:**
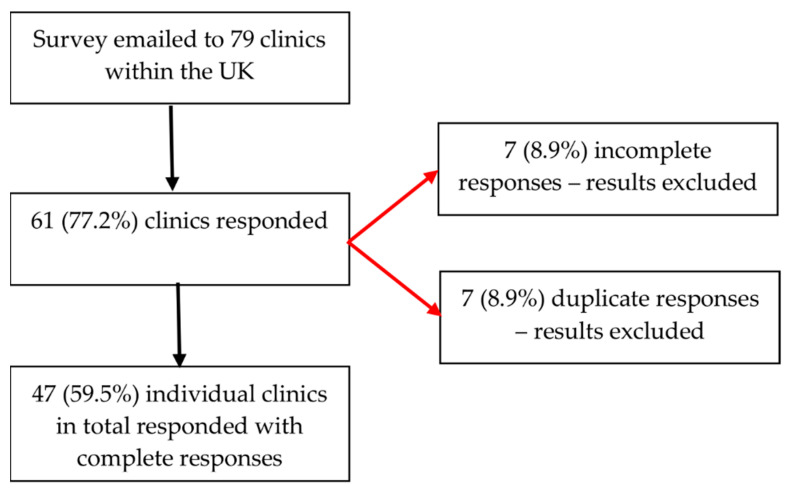
Flowchart of survey respondents.

Sixty-one out of the 79 clinics responded, fourteen responses were excluded (seven incomplete and seven duplicate), leaving the final number of responses analysed as 47 (Figure 1). 

### 3.1. Demographics of the Units

Table 1 outlines the demographic data of the units that responded to the survey. It demonstrates that the majority of practices treat both NHS and privately funded patients (36, 77%), base their ET success rate on CPR (27, 57%) and estimate their LBR to be between 30 and40% (28, 60%).

### 3.2. Embryo Transfers

Seven clinics (15%) allowed individuals to utilise their preferred ET technique. No zygote intrafallopian transfers were performed by any of the clinics (Table 2). 

When the published HFEA clinic success rates were considered, those clinics performing more transfers appeared to have better LBR than those performing less ET’s (Table 3).

### 3.3. ET Preparation

Most units did not use sedation for ET (94%), with one unit (2%) using sedation when required (when a patient was unable to tolerate the procedure without sedation). Forty-three (91%) of the clinics cleaned the cervix prior to ET and 33 (72%) removed cervical mucus with a cotton wool swab (Table 4). Most units (78%) would abandon the ET if there was fluid within the endometrial cavity on ultrasound. Thirty-nine (83%) of the clinics performed ultrasound-guided ET with nursing staff performing the majority of the ultrasound scanning (92%).

### 3.4. ET Technique

The most common ET technique was the afterload technique (53%), with 100% of respondents using soft catheters (Table 5). Clinics generally used (72%) a stylet for less than 25% of their transfers and the routine use of tenaculum was uncommon. Most (91%) reported deposition of the embryo in the upper or middle portion of the uterine cavity, although exact deposition points from the uterine fundus varied from 0.5 cm to over 2 cm. Embryo retention following transfer was <5% in all clinics, with 31 respondents (66%) re-transferring the embryo in a new catheter when this occurred.

**Figure 2:**
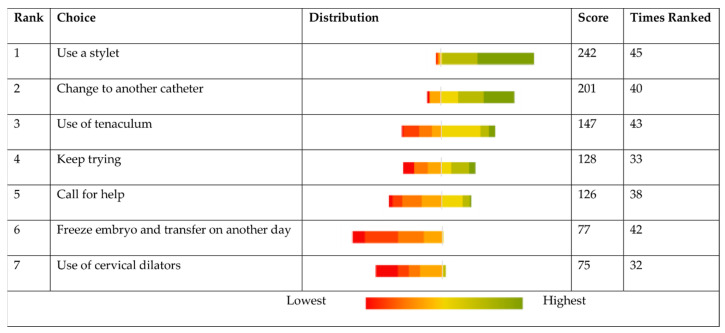
If there is difficulty in ET, what would be your preferred options in order 1–7.

Clinics were asked to rank how they would deal with a difficult transfer and what steps they would take (Figure 2). When faced with a difficult transfer, the majority responded claiming to use a stylet. Use of cervical dilators was the most infrequent response.

**Figure 3:**
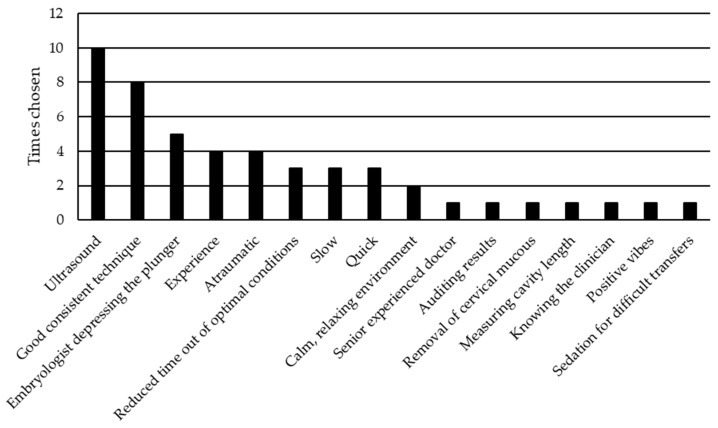
Most important aspects of embryo transfer.

When the respondents were asked what they thought was the most important aspect with regard to ET, the majority of responses suggested guidance with ultrasound and good consistent technique (Figure 3). Interestingly, there were three responses stating that a slow steady transfer improves chances of success, whilst three other responses urged speedier transfers. 

When comparing the LBR published by the HFEA for units, very similar results were observed between those units that used ultrasound guidance and those which used clinical touch technique (CTT). For the CTT, the LBR was 22.8% (SD ± 3.06) compared to 22.4% (SD ± 5.4) for the ultrasound-guided group (*p* = 0.873). 

## 4. Discussion

This contemporary national survey updates the 16-year-old previous survey on ET technique in the UK and highlights the existing wide variation in practice with no standardised approach to the procedure prevailing in the UK. It therefore emphasises the urgent need for a standardised national protocol to ensure best outcomes for women undergoing IVF in the UK [22]. 

Over the years there have been many changes in ET techniques in general, with new evidence demonstrating the benefit of particular practices to improve outcome, such as the use of ultrasound guidance [1], soft catheters [14,17,26] and avoiding prolonged bed rest following transfer [28]. Reassuringly, the majority of units that responded, appeared to acknowledge the new evidence in their practice (83% ultrasound guidance, 100% soft catheters and 68% immediate mobilisation). Interestingly, we unexpectedly found no significant difference in LBR between clinics regardless of the use of ultrasound guidance. 

**Figure 4:**
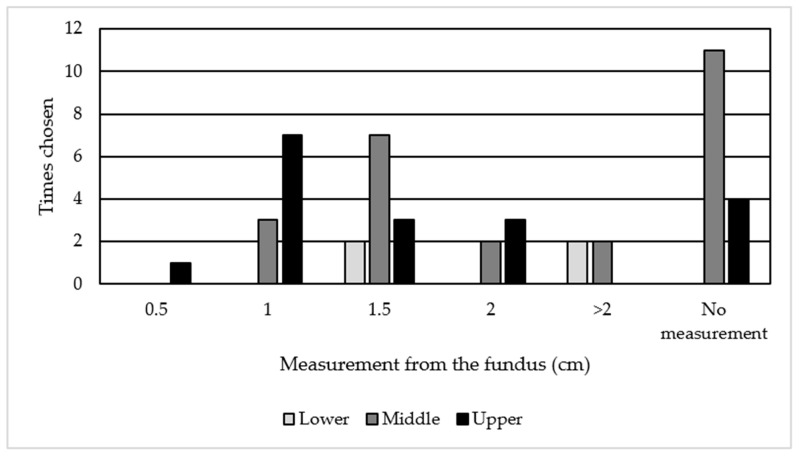
Units’ interpretation on distance from fundus to location in uterine cavity.

Positioning of the embryo catheter in the upper or middle third of the cavity was the practice in 91% of the units, in line with the systematic reviews [14,17]. However, this apparently excellent practice should be considered with caution since some survey responders appear to have different interpretations of the terms upper, middle and lower third of the cavity (Figure 4). They determined the upper third of the cavity as 0.5→ 2 cm, middle third as 1 → 2 cm and the lower third as 1.5 → 2 cm from the fundus. Among those respondents who measured the distance from the fundus, 85% placed the catheter 1–2 cm from the fundus of the uterus. Frequency of depositing the embryo at the upper third of the cavity increased to 97% if we included those who transfer at >2 cm in keeping with the recommendations from the Cochrane reviews [14,17,29]. This draws attention to the need for clarity in a future guideline/study protocol in which embryo deposition is described.

Despite the available evidence supporting immediate withdrawal of the catheter following embryo expulsion [14,17,30,31] only six units (13%) adhered to this, with the remaining units allowing a delay prior to removal. There was no significant difference in pregnancy rates between the groups regardless of this practice [30,31]. However this practice may unnecessarily prolong the uncomfortable procedure for the patient without conferring any benefit. 

All units reported embryo retention rates at <5% in keeping with previously quoted incidence rates [8]. Maintaining a low retention rate would help reduce patient anxiety and reduce the time that the embryo is outside of the incubator optimal conditions. Prolonged transfer times are known to have a detrimental effect on pregnancy rates [32,33], although the retransfer of retained embryos has not shown to be detrimental [34,35,36,37,38]. 

Conversely, there are areas with room for improvement. Amongst them, of concern is how clinics approach fluid within the endometrial cavity, since 21% of respondents claimed that they would either aspirate (15%) or would proceed with transfer (6%) when there was fluid identified within the endometrial cavity, despite available advice to the contrary [8,39]. We appreciate that fluid in the endometrial cavity is not an absolute contraindication to ET, and that in cases where embryos need to be refrozen this may have a negative impact on subsequent implantation and LBR [40]. Other studies have also found that those with transient, small amounts of fluid within the endometrial cavity (<3.5 mm) are not associated with poorer outcomes [41,42]. However, those with known hydrosalpinx, or with persistent endometrial fluid in the cavity, continue to have poorer outcomes compared to those without fluid in the endometrial cavity [42]. These cases need to be dealt with on an individual basis, taking into account patient preference whilst weighing the risks and benefits of continuing with the ET. The recommendation from our survey would be to abandon the ET if endometrial fluid is found in the cavity and freeze the embryo for transfer in a subsequent cycle, particularly since emerging evidence is showing no detrimental effect when embryos are refrozen [43,44]. 

The frequent use of a tenaculum in some units is another concern. The use of a tenaculum is not only painful but can also have a negative impact on embryo implantation rates due to increased uterine contractions due to stimulating oxytocin release [45,46]. With this in mind, the use of a tenaculum for ET should only be used once all other options are exhausted, yet surprisingly, it was the third most popular option to be used for difficult transfers. When 57% of respondents reported having never used a tenaculum or only having used one several times in their career, this raises the question how much their technique differs to those who use the tenaculum on a more frequent basis. 

One other interesting feature identified in our survey was that the majority of respondents estimated their LBR to be between 30 and 40%. However, the 2017 HFEA data reported most of the clinics having a LBR between 20 and 30% [47]. Although it is possible that this is due to the HFEA data being two years older than when the clinics responded to our survey, this may also be relevant to personal perception versus actual figures, and further highlights the important impact such discrepancies may have when patients are counselled by the clinicians in these units. Relevant to this, CPR was the preferred marker of success for the responders, since presumably it is an easily and relatively rapidly attained marker of success, with the majority of clinics performing the initial scan themselves to confirm a pregnancy, and thereby acquiring this data [48]. Subsequently, patients may be lost to follow up, and accurate LBR data is more difficult to collate [49]. Importantly, LBR is a mandatory outcome to be reported in the UK, and possibly the most relevant data for patients. However, publicizing the CPR, which is naturally higher than the LBR, may be more attractive to patients [48]. 

Whilst there are a number of questions where concordance was observed in this survey, there were more responses that differed than were similar. This lack of standardisation amongst units can be one of the reasons why LBR between clinics range from 11 to 34% [47]. We appreciate that there are numerous other steps involved in the ART technique that impact overall success rates, including type of ovarian stimulation cycles, oocyte retrieval and laboratory techniques. However, if standardisation of ET techniques were to occur, it could potentially highlight other imperfect areas in the above-mentioned steps of the IVF process that also have an impact on the LBR.

Standardization could also reduce research bias, which has previously been noted by Gambadauro et al. [50]. When reviewing published trials in IVF there was very little information about the methods and execution involved in the ET and this could potentially be a source of performance bias [50,51].

Our findings are in agreement with a previous survey conducted by the ASRM [4], which also highlighted the need for standardization. They also demonstrated a highly diverse approach to the ET technique, with multiple areas of discordance including use of a patient relaxant at the time of ET, direction of catheter removal and duration of bed rest following transfer [4]. As a consequence of their survey, the ASRM have been able to produce a protocol for ET suitable for North American practice [4,17,52]. We anticipate our survey should facilitate the launch of a similar national/European protocol following discussion with representative bodies such as the British fertility society (BFS) and/or the European society of human reproduction and embryology (ESHRE).

## 5. Recommendation

The previously mentioned ASRM survey [4], as well as the review by Saravelos et al. [14], made recommendations based on their literature reviews. These can be seen in Table 6.

Based on the findings of this survey, and the above evidence, we propose the following approach to embryo transfer:

1. No routine use of anaesthesia or analgesia.
2. Use sterile gloves.
3. No use of warmed speculum.
4. Use sterile water or normal saline for speculum lubrication.
5. Clean the cervix with normal saline or laboratory media.
6. Use cotton wool or gauze to clean the cervix and remove mucus.
7. Use ultrasound guidance for embryo transfer.
8. Abandon transfer if fluid is within the endometrial cavity.
9. Perform mock transfer for specific indication.
10. Afterload technique.
11. Deposit the embryo in the upper/middle portion of the endometrial cavity.
12. Use a stylet when required or anticipated difficulty.
13. Avoid the use of tenaculum/vulsellum.
14. Slow and steady pressure of plunger.
15. Remove the catheter either straight or rotational immediately following transfer.
16. Immediate ambulation.

The main limitation of this survey was that we did not achieve full coverage of all UK IVF units. The response rate was reasonably high (59%), but we accept that this survey is not necessarily representative of universal practice within the UK. The main instrument utilised to gather information in our study was a questionnaire. We specifically developed this questionnaire with the involvement of a number of specialists and experts from around the UK to provide a snapshot of current practice, and it was not for general use among the public. Therefore, although we acknowledge that not validating this questionnaire as a limitation of our work, we followed similar pathways to other previous surveys [4,6,53,54] in this field, and the involvement of multiple experts in the field in its development improves its validity. The data obtained is qualitative and should be interpreted as such, but it is meant to highlight the variations in current practice within the UK and to prompt conversations on how standardisation could be achieved in ET techniques. 

The strengths of this survey are that it is the first of its kind in the UK, and comprehensively and systematically dissects out the practice of ET procedures. It emphasized the concordance, discordance and areas of improvement required in certain practices involved in the ET process, identifying the areas in need of a standardized approach. Areas of improvement should aim to abandon ET when fluid is seen in the endometrial cavity and only use tenaculums when all other options have been exhausted. 

ET techniques have been shown to have a significant impact on pregnancy rates [24,27,55] and the variation between practices could have an influence (along with other factors of the IVF process) on a unit’s success rate. In a field of medicine where every percentage point counts, slight changes could result in significant improvement in success rates and patient satisfaction. Therefore, we have a responsibility to ensure that all patients receive best evidence-based care, and this survey brings to light that this may not be the case, at least in some aspects of the ET process in the UK.

## 6. Conclusions

This is the first survey that sheds light on contemporary practice and attitudes among different units regarding ET in the UK. It highlights the urgent need for standardisation in ET, a process that is vital for IVF success rates. Such standardisation of practice will facilitate practitioner training, research and ultimately IVF success rates. The lack of evidence for best practices that prevails in many areas of the ET procedure will need to be overcome with a consensus expert meeting and review of all literature. We believe that areas of discordance identified in our survey, where there is insufficient evidence for the most favourable method, will guide future research to fill the gaps in our current knowledge.

## Figures and Tables

**Table 1:** Unit demographics.

**Types of IVF Practice *n*** (**%**)	
NHS	2 (4)
NHS and Private	36 (77)
Private	9 (19)
**Basis of ET success *n* (%)**	
Positive pregnancy test	13 (28)
Clinical pregnancy rate	27 (57)
Live birth rate	5 (11)
No response	2 (4)
**Persons performing the ET *n* (%)**	
Consultant only	18 (38)
Consultant and nurse	14 (30)
Consultant, registrar and nurse	7 (15)
Consultant and registrar	6 (13)
Nurse only	2 (4)
**Estimated clinical pregnancy rates per ET *n* (%)**	
20–30	3 (6)
30–40	18 (38)
40–50	23 (49)
50–60	1 (2)
60–70	0 (0)
>70	1 (2)
No response	1 (2)
**Estimated Live birth rate per ET *n* (%)**	
20–30	13 (28)
30–40	28 (60)
40–50	3 (6)
50–60	0 (0)
60–70	1 (2)
No response	2 (4)

**Table 2:** Number of transfers performed by units.

**Presence of Standardised Technique within the Unit *n*** (**%**)	
Standard technique	40 (85)
Technique based on individual preference	7 (15)
**Number of ETs per year *n* (%)**	
<500 *n* (%)	7 (15)
500–1000 *n* (%)	20 (43)
1000–1500	10 (21)
1500–2000	2 (4)
>2000	8 (17)
**Number of transmyometrial transfers per year *n* (%)**	
10	1 (2)
5	2 (4)
3	1 (2)
2	7 (15)
1	6 (13)
0	30 (64)

**Table 3:** Number of ETs relating to average HFEA LBR.

Number of ETs	Number of Clinics	Average HFEA LBR (%)
<500	7	20.1
500–1000	20	22.8
1000–1500	10	22.2
1500–2000	2	28.5
>2000	8	24.3

**Table 4:** Patient and practitioner preparation prior to ET.

**Patient Relaxant *n*** (**%**)	
None	44 (94)
Voltarol	1 (2)
Sedation when required	1 (2)
Sedation	1 (2)
**Sterility of Procedure *n* (%)**	
Sterile gloves after handwashing	27 (57)
Aseptic technique	18 (38)
Scrubbed and gowned	2 (4)
**Warmed speculum *n* (%)**	
Yes	11 (23)
No	36 (77)
**Lubrication on speculum *n* (%)**	
None	10 (21)
Culture media	1 (2)
Normal Saline	23 (49)
Sterile water	11 (23)
Ultrasound gel	2 (4)
**What is used to clean the cervix *n* (%)**	
Normal Saline	34 (72)
Media from lab	7 (15)
Not cleaned	4 (9)
Sterile water	2 (4)
**Instrumentation used to clean the cervix *n* (%)**	
Cotton wool	23 (50)
Gauze sponge on forceps	19 (41)
Cotton wool and Gauze	2 (4)
Pipette	1 (2)
N/A	1 (2)
**Removal of endocervical mucous *n* (%)**	
Cotton wool	29 (63)
Aspirate	4 (9)
Cotton wool and flush	4 (9)
Flush	2 (4)
Not removed	7 (15)
**Embryo transfer technique *n* (%)**	
2D ultrasound guidance	38 (81)
3D ultrasound guidance	1 (2)
Clinical touch technique	7 (15)
Dummy ET and measurement of cavity length	1 (2)
**Person performing the ultrasound scan *n* (%)**	
HCA	8 (17)
Embryologist	1 (2)
Nurse	36 (77)
Doctor	4 (9)
Ultrasound technician	1 (2)
**Approach to fluid within the endometrial cavity *n* (%)**	
Abandon the transfer	35 (74)
Aspirate the fluid and continue with transfer	7 (15)
Continue with the transfer	3 (6)
No response	2 (4)
**Use of a routine mock transfer *n* (%)**	
For specific indication	27 (57)
Not routinely done	10 (21)
Immediately before transfer	4 (9)
At oocyte retrieval	2 (4)
Before cycle begins	4 (9)

**Table 5:** ET technique.

**Embryo Transfer Technique** (***n*%**)	
Afterload technique	24 (53)
Trial with transfer technique	12 (27)
Direct technique	9 (20)
**ET catheter preference *n* (%)**	
Wallace	29 (62)
Cook	22 (47)
Kitazato	6 (13)
Surepro	2 (4)
Labotect	1 (2)
**Use of stylet *n* (%)**	
All the time	1 (2)
>50% of transfers	6 (13)
25–50% of transfers	5 (11)
<25% of transfers	34 (72)
Never	1 (2)
**Use of a tenaculum *n* (%)**	
Never	9 (19)
Several times in career	18 (38)
<10% of transfers	18 (38)
<30% of transfers	2 (4)
**Approximate location of catheter tip in uterine cavity *n* (%)**	
Upper third	18 (38)
Middle third	25 (53)
Lower third	4 (9)
**Approximate distance embryo is is deposited (cm) from uterine fundus *n* (%)**	
0.5	1 (2)
1	10 (21)
1.5	12 (26)
2	5 (11)
>2	4 (9)
Don’t measure	15 (32)
**Who depresses the plunger once the catheter is in place *n* (%)**	
Clinician	34 (72)
Embryologist	13 (28)
**Speed and process of embryo deposit *n* (%)**	
As slowly as possible	7 (15)
Slow pace with steady pressure	29 (62)
Moderately fast with steady pressure	11 (23)
As quick as possible	1 (2)
**Approach to retained embryos *n* (%)**	
Retransfer in same catheter	19 (40)
Retransfer in new catheter	31 (66)
**Frequency of retained embryos *n* (%)**	
<1% of ET	35 (74)
1–5%	12 (26)
**Presence of blood or mucus on catheter tip *n* (%)**	
<5%	22 (47)
5–10%	18 (38)
10–20%	5 (11)
20–30	2 (4)
**Duration catheter left inside cavity following embryo deposition *n* (%)**	
Immediately removed	6 (13)
5–10 s	18 (38)
10–20 s	17 (36)
30 s	5 (11)
1 min	3 (6)
**Direction catheter removed *n* (%)**	
Straight	21 (45)
Rotate as removed	25 (53)
Both	1 (2)
**Patient remaining supine after transfer *n* (%)**	
Get up immediately	32 (68)
5–10 min	15 (32)

**Table 6:** ET recommendations.

Recommendation	ASRM Guideline [4]	Saravelos et al. [14]
Removal of cervical mucous	Grade B evidence	Grade B evidence
Use soft ET catheters	Grade A evidence	Grade A evidence
Abdominal ultrasound guidance	Grade A evidence	Grade A evidence
Embryo transfer to central or upper cavity	Grade B evidence	Grade B evidence
Immediate catheter withdrawal	Grade B evidence	Grade B evidence
Immediate ambulation	Grade A evidence	Grade A evidence
Immediate retransfer of retained embryo	Grade B evidence	Grade B evidence

## Data Availability

The data presented in this study is available on request from the corresponding author. The data is not publicly available due to privacy.

## Supplementary Materials

The following are available online at https://www.mdpi.com/article/10.3390/jcm10132839/s1, Figure S1: Final Survery.
