# Supplementary material for: National Survey Highlights the Urgent Need for Standardisation of Embryo Transfer Techniques in the UK
Source: J Clin Med. 2021 Jun 27;10(13):2839. doi: 10.3390/jcm10132839 (PMC8267796; doi:10.3390/jcm10132839)
Supplement: Supplementary file 1 [file jcm-10-02839-s001.zip › jcm-1223039-SI.pdf]

**Supplementary Figure 1 –** Final Survey

Demographics

Name of Organisation- _________________

Types of IVF practice- NHS / Private / NHS and private

How many embryo transfers do you do each year - <500 / 500<1000 / 1000<1500 / 1500<2000 / >2000

How many ZIFT transfers do you do per year? _________________

How many transmyometrial transfers do you do each year? _________________

Basis of ET success rate - Positive hCG / Clinical pregnancy rate / Live birth rate

Estimated Success rate (%)- <20 / 20-30 / 30-40 / 40-50 / 50-60 / 60-70 / >70

Estimated clinical pregnancy rate (%)- <20 / 20-30 / 30-40 / 40-50 / 50-60 / 60-70 / >70

Estimated live birth rate (%)- <20 / 20-30 / 30-40 / 40-50 / 50-60 / 60-70 / >70

Who performs the embryo transfer (ET) – Nurse / Registrar / Consultant

Patient and practitioner preparation

Presence of a standard technique/operating procedure (SOP) for ET practice in unit- Standard technique / Technique based on individual preference

Use of patient relaxant- No / Gas and air / Sedation / Nifedipine / Ritodrine / Other

Sterility of the procedure - Sterile gloves after handwashing / Aseptic technique / Scrubbed and gowned

Use of warmed speculum – Yes / No

Lubrication used on speculum – None / Culture media / Normal saline / Sterile water / Ultrasound gel

What do you use to clean the cervix before transfer - Normal saline / Media from lab / Other / NA(don't cleanse)

What Instruments do you use to clean cervix? Cotton swab / Gauze sponge on forceps / NA / Other

How do you remove mucous from the endocervical canal - Cotton swab / Flush / Cotton swab and flush / Aspirate / NA

What technique is used in your clinic for embryo transfer? Clinical touch technique / 2D Abdominal ultrasound / 3D Abdominal ultrasound / Other (please specify)

Person performing US guidance - US technician / Nurse / Doctor / NA / Other

Approach to fluid in the cavity- Cancel transfer / Aspirate fluid / Continue with transfer

Routine performance of mock transfer - Before cycle begins / During stimulation / At oocyte retrieval / Immediately before transfer / Not routinely done / For specific indication

Embryo transfer technique

Predominant technique - Trial with transfer technique / Afterload technique / Direct technique

ET catheter preference – Cook / Wallace / Other

If there is difficulty in ET, what would you be your preferred options in order 1-7

- Use a stylet
- Change to another catheter
- Use of tenaculum
- Freeze embryo and transfer on another day
- Call for help
- Keep trying
- Use of cervical dilators

Use of stylet - All the time / >50% / 25-50% / <25% / Never

Frequency of transfers using a tenaculum – Never / Several times in career / <10% / <30% / <50%

Approximate location of endometrial cavity where tip of catheter is aimed - Upper third / Middle third / Lower third

Distance from uterine fundus embryo deposited - 0.5cm / 1cm / 1.5cm / 2cm / >2cm / Don't measure

Who pushes the plunger once the embryo catheter is in place – Clinician / Embryologist

Speed and process of plunge - As slowly as possible / Slow with steady pressure / Moderately fast with steady pressure / As quick as possible
Approach to retained embryos - Retransfer in same catheter / Reload into new catheter
Frequency of retained embryos (%) - <1 / 1-5 / 5-15 / 15-20 / >20
Frequency of blood or mucous on end of catheter(%) - <5 / 5-10 / 10-20 / 20-30 / 30-40 / >40
Time after embryos expelled into cavity before removing catheter - Immediate removal / 5-10sec /10-20sec / 30 sec / 1min / Other
Direction for catheter removal – Straight / Rotate as removed
Patient remains supine after transfer - Gets up immediately / 5-10 min / 10-15 min / 15-30 min / >30min

Please highlight those areas that you think are the most relevant to your practice. __________________
